# Caspase Recruitment Domain Family Member 8: A Favorable Target in the Pathogenesis of Atherosclerosis

**DOI:** 10.31083/RCM44518

**Published:** 2026-01-21

**Authors:** Dandan Tian, Li Liu, Guang-Gui Zeng, Jinrong He, Huiqin Liu, Dandan Ma, Zixin Yang, Xiangyan Ma, Yunxiang Cao, Chunyan Xu

**Affiliations:** ^1^Department of Intensive Care Medicine, Hunan University of Medicine General Hospital, 418000 Huaihua, Hunan, China; ^2^Department of Nursing, Hunan University of Medicine General Hospital, 418000 Huaihua, Hunan, China; ^3^School of Medicine, Shanghai Jiao Tong University, 200127 Shanghai, China; ^4^Department of Cardiology, Hunan University of Medicine General Hospital, 418000 Huaihua, Hunan, China

**Keywords:** *CARD8*, atherosclerosis, cardiovascular disease, therapeutic potential, molecular mechanisms

## Abstract

Atherosclerosis, a lipid-driven chronic inflammatory disease, is the primary pathological basis of cardiovascular diseases, characterized by endothelial injury, lipid deposition, immune cell infiltration, and chronic inflammation. The NOD-like Receptor Pyrin Domain-Containing 3 (*NLRP3*) inflammasome has emerged as a crucial mediator of inflammation in atherosclerosis, with caspase recruitment domain family member 8 (CARD8) acting as a key regulatory component. Indeed, CARD8, a member of the caspase recruitment domain family, regulates immune responses by modulating inflammasome activity, particularly NLRP3. Recent studies suggest that CARD8 influences various aspects of atherosclerotic development, including lipid accumulation, oxidative stress, vascular inflammation, smooth muscle cell proliferation, and plaque instability. Thus, this review summarizes the latest findings on the role of CARD8 in the pathogenesis of atherosclerosis, with a focus on the regulatory effects of this component on immune cells and inflammatory pathways. We also discuss the potential of targeting CARD8 as a therapeutic strategy for atherosclerosis, exploring the current preclinical and clinical evidence.

## 1. Introduction

Atherosclerosis is a complex, multifactorial disease that is characterized by the chronic accumulation of lipids, inflammatory cells, and extracellular matrix within the arterial wall [1]. This disease is a leading cause of cardiovascular morbidity and mortality worldwide, contributing to major conditions such as coronary artery disease (CAD), stroke, and peripheral artery disease [2, 3]. The pathogenesis of atherosclerosis involves a series of processes, including endothelial injury, lipid accumulation, smooth muscle cell migration, and the formation of atherosclerotic plaques [4, 5]. Despite the widespread use of statins and other lipid-lowering therapies, the incidence of cardiovascular events remains high, and many patients still experience recurrent cardiovascular events even after treatment, underscoring the need for more effective and targeted therapies to address the underlying disease processes. Historically, atherosclerosis was primarily considered a disease of lipid accumulation; however, recent research has revealed that immune cells play a pivotal role in the development and progression of the disease [6]. The immune response in atherosclerosis is initiated by the retention of low-density lipoprotein (LDL) particles in the arterial intima, which undergo oxidative modifications [7]. These modified lipoproteins are recognized by immune cells, primarily macrophages, which engulf them to form foam cells [8]. Foam cell formation, in turn, triggers a cascade of inflammatory events that contribute to plaque instability [9, 10, 11, 12]. Furthermore, T lymphocytes and dendritic cells, which infiltrate the atherosclerotic lesions, release pro-inflammatory cytokines and chemokines, thus perpetuating the inflammatory response and contributing to lesion progression [13, 14, 15, 16, 17, 18]. It is now well-established that inflammation within the arterial wall is a central driver of atherosclerosis and that modulation of the immune response may offer potential therapeutic avenues for treatment [19]. Chronic low-grade inflammation in atherosclerosis is not only responsible for plaque formation but also for the destabilization of plaques, leading to acute cardiovascular events such as myocardial infarction and stroke [20]. Therefore, understanding the mechanisms that regulate immune responses within atherosclerotic plaques is critical for the development of novel therapies aimed at controlling inflammation and stabilizing plaques.

Inflammasomes are multi-protein complexes that are formed in response to cellular stress and injury [21, 22, 23, 24]. These complexes play a crucial role in innate immunity by sensing pathogens, danger signals, and cellular damage. The NOD-like Receptor Pyrin Domain-Containing 3 (*NLRP3*) inflammasome, one of the best-studied inflammasomes, is composed of the sensor protein *NLRP3*, the adapter protein ASC, and the effector protein caspase-1 [25, 26, 27, 28]. Upon activation, *NLRP3* inflammasome triggers the processing and release of pro-inflammatory cytokines, particularly Interleukin (IL)-1β and IL-18, which are involved in the promotion of inflammation and the recruitment of immune cells to the site of injury [29, 30, 31, 32]. In the context of atherosclerosis, *NLRP3* inflammasome activation plays a central role in the development of inflammatory responses within the atherosclerotic plaques [33, 34]. Studies have shown that *NLRP3* inflammasome activation in macrophages, endothelial cells, and smooth muscle cells contributes to the local inflammatory environment that accelerates plaque progression and destabilization [35, 36, 37, 38]. For example, the release of IL-1β from activated macrophages leads to further recruitment of immune cells, which exacerbates the inflammatory response and promotes the formation of necrotic cores in the plaque. As a result, inhibiting *NLRP3* inflammasome activity has emerged as a promising therapeutic strategy for reducing inflammation and preventing plaque rupture in atherosclerosis.

Caspase Recruitment Domain Family Member 8 (*CARD8*) is a recently identified member of the caspase recruitment domain (CARD) family of proteins that has been shown to play an important role in the regulation of inflammasome activation [39, 40, 41]. *CARD8* is highly expressed in immune cells such as macrophages, dendritic cells, and neutrophils, where it interacts with the *NLRP3* inflammasome to modulate its activation [42, 43, 44]. Unlike other inflammasome components that promote inflammasome activation, *CARD8* acts as a negative regulator by inhibiting the excessive activation of *NLRP3* [45, 46, 47]. *CARD8* achieves this through interactions with *NLRP3* and caspase-1, preventing the activation of the inflammasome under basal conditions. In addition to its role in inflammasome regulation, *CARD8* is also involved in the modulation of other immune pathways, such as apoptosis and the regulation of cytokine production [42, 48]. The dual role of *CARD8* in immune regulation has made it an attractive candidate for further investigation in the context of atherosclerosis. Some studies suggest that *CARD8* expression is upregulated in macrophages within atherosclerotic lesions, where it helps control the inflammatory response by inhibiting excessive inflammasome activation [49, 50, 51, 52]. However, the precise role of *CARD8* in atherosclerosis remains unclear, as some studies have also suggested that *CARD8* may have pro-inflammatory effects under certain conditions, particularly in the context of macrophage activation and foam cell formation. Given these conflicting findings, further research is needed to elucidate the full spectrum of *CARD8*’s function in atherosclerotic disease and its potential as a therapeutic target.

In recent years, the exploration of *CARD8* as a therapeutic target in atherosclerosis has gained attention. Targeting *CARD8* could potentially modulate inflammasome activation, reduce inflammation, and stabilize atherosclerotic plaques [43, 51, 53, 54]. The development of small molecules or biologics that can selectively target *CARD8* expression or its interactions with inflammasome components holds great promise for therapeutic intervention in atherosclerosis. However, the clinical translation of *CARD8*-based therapies faces several challenges. For instance, the specificity and selectivity of *CARD8*-targeting agents need to be carefully evaluated to avoid unintended effects on other immune pathways. Additionally, the potential for off-target effects and the long-term safety of such therapies must be addressed before clinical application. Despite these challenges, the promise of *CARD8* as a therapeutic target in atherosclerosis underscores the need for further research to refine and optimize strategies that target this protein. This review aims to provide a comprehensive overview of the current understanding of *CARD8*’s role in atherosclerosis, with a focus on its function in regulating the *NLRP3* inflammasome and its impact on the inflammatory processes driving atherosclerotic disease. We will examine the molecular mechanisms by which *CARD8* influences immune cell activation and cytokine production, as well as its potential as a therapeutic target in atherosclerosis. Additionally, we will discuss current research on *CARD8*-targeted therapies and explore the challenges and opportunities for translating these findings into clinical practice.

## 2. Literature Search and Methodology

Web of Science is a globally authoritative retrieval system operated by Clarivate, widely cited and utilized in the academic community. It boasts an extensive subject coverage, encompassing 256 disciplines across diverse fields such as science and technology, social sciences, arts, and humanities, serving as a comprehensive resource library. Its literature resources are remarkably abundant: as of December 2024, the Web of Science Core Collection has included 79 million records, with the total number of records across the entire platform reaching 171 million. Additionally, the system contains citation information for each article.

Web of Science excels in retrieval functionality, featuring a variety of retrieval methods and rules. It also possesses unique citation indexes, in-depth analysis tools, academic influence evaluation functions, and personalized services. These capabilities provide support to researchers in multiple research aspects, such as understanding the development context of research topics, assessing influence, and tracking the progress of research projects.

**Fig. 1.**
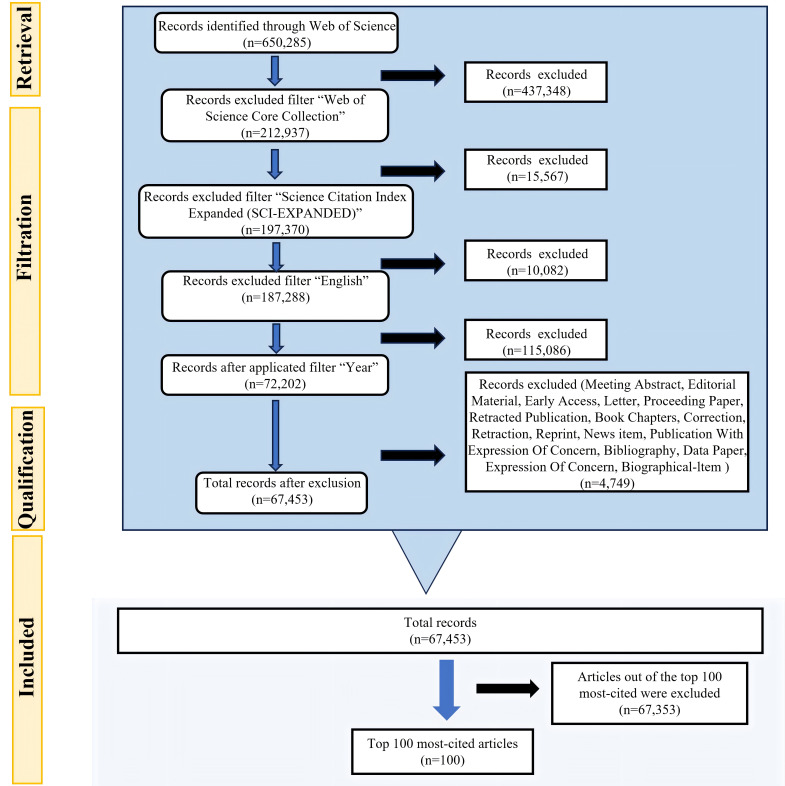
**Flowchart of the search methodology and data source**.

Our team used the Web of Science Core Collection as the data source, and the configured retrieval formula was “(ALL = *CARD8*) OR (ALL = Caspase Recruitment Domain Family Member 8)”. Through this retrieval process, a total of 603 pieces of literature were obtained, including 491 research articles, 76 reviews, and 36 pieces of literature of other types. To enhance the accuracy of the content, all literature has been downloaded, and a comprehensive full-text review has been conducted. The flow chart of the screening process is shown in Fig. 1.

## 3. The Immunological Landscape of Atherosclerosis

### 3.1 Pathogenesis of Atherosclerosis: From Endothelial Dysfunction to Plaque Formation

Atherosclerosis is a complex and progressive vascular disease characterized by the accumulation of lipids, inflammatory cells, and extracellular matrix components within the arterial wall [55, 56, 57, 58, 59, 60]. The development of atherosclerosis is initiated by endothelial injury, which promotes the retention and oxidation of LDL in the subendothelial space [61, 62, 63, 64]. This injury disrupts the homeostasis of the arterial wall and triggers a series of pathological events, including lipid deposition, smooth muscle cell proliferation, and immune cell infiltration [65, 66, 67, 68, 69, 70]. The retention of oxidized LDL (ox-LDL) in the intima activates endothelial cells, which secrete pro-inflammatory cytokines and adhesion molecules that recruit circulating monocytes to the site of injury [63, 71, 72]. Once monocytes are recruited, they differentiate into macrophages, which engulf ox-LDL and transform into foam cells [73, 74]. These foam cells contribute to the formation of a lipid-rich core within the plaque. As the plaque matures, smooth muscle cells migrate into the intima, forming a fibrous cap that stabilizes the plaque. However, in the presence of sustained inflammation, the plaque can become unstable, leading to rupture and thrombosis, which is a major cause of acute cardiovascular events such as myocardial infarction and stroke [75, 76].

Recent research has elucidated the crucial role of immune responses in the pathogenesis of atherosclerosis [68, 77]. The inflammatory process is driven by the activation of innate and adaptive immune cells, which orchestrate the recruitment and activation of additional inflammatory mediators. Central to the regulation of inflammation in atherosclerosis are the inflammasomes, multiprotein complexes that mediate the activation of pro-inflammatory cytokines such as IL-1β and IL-18 [78, 79]. Among the different inflammasomes, the *NLRP3* inflammasome is considered one of the most important in atherosclerosis, as its activation is implicated in the chronic inflammation that accelerates plaque progression [80, 81].

### 3.2 Immune Cells and Their Roles in Atherosclerosis

Immune cells play a central role in the development and progression of atherosclerosis [82]. The disease is marked by the infiltration of various immune cell types, including monocytes, macrophages, dendritic cells, T lymphocytes, and B lymphocytes, all of which contribute to the inflammatory microenvironment of the atherosclerotic plaque [83, 84, 85, 86, 87, 88].

Monocytes and macrophages are the most prominent immune cells involved in atherosclerosis [84, 89]. In response to endothelial damage and lipid accumulation, monocytes migrate to the plaque and differentiate into macrophages [90, 91]. These macrophages, in turn, become foam cells by ingesting ox-LDL, contributing to the formation of the lipid-rich core of the plaque [73, 92]. Foam cells also release a range of pro-inflammatory cytokines, such as IL-1β, IL-6, and Tumor Necrosis Factor (TNF)-α, which further propagate the inflammatory response and promote plaque growth [93, 94]. Moreover, the presence of macrophages and foam cells in the plaque is associated with increased oxidative stress, which exacerbates endothelial dysfunction and amplifies the inflammatory cycle [95, 96]. T lymphocytes, particularly CD4^+^ T helper cells, are also critical in the immune response to atherosclerosis [97]. Th1 cells, which produce Interferon (IFN)-γ, promote the activation of macrophages and the secretion of pro-inflammatory cytokines, thereby enhancing plaque inflammation [98, 99]. Conversely, regulatory T cells (Tregs) exert an anti-inflammatory effect by secreting cytokines such as IL-10, which dampens the immune response and promotes plaque stability [100, 101]. An imbalance between pro-inflammatory Th1 cells and anti-inflammatory Tregs can lead to the destabilization of atherosclerotic plaques, increasing the risk of rupture [87, 102]. Additionally, the contribution of dendritic cells to atherosclerosis has become an area of increasing interest. Dendritic cells are important antigen-presenting cells that activate T lymphocytes, influencing the adaptive immune response within the plaque [103, 104]. They have been shown to promote both pro-inflammatory and anti-inflammatory responses depending on their activation state, and their role in plaque development and progression remains an active area of investigation.

### 3.3 Inflammation and Inflammasomes: Central to Atherosclerosis Pathogenesis

The inflammatory process in atherosclerosis is tightly regulated by several signaling pathways, with inflammasomes playing a pivotal role in modulating the immune response [105]. Inflammasomes are large, multi-protein complexes that respond to pathogen- or damage-associated molecular patterns and activate the pro-inflammatory cytokines IL-1β and IL-18 [21]. Among the various inflammasomes, the *NLRP3* inflammasome has been extensively studied in the context of atherosclerosis due to its critical involvement in both innate immunity and the chronic inflammation seen in atherosclerotic lesions [80, 106]. Upon activation, the *NLRP3* inflammasome triggers the maturation of IL-1β and IL-18, which further amplify the inflammatory cascade by promoting immune cell recruitment, activation, and cytokine production [107]. Elevated levels of IL-1β in the plaque microenvironment have been associated with increased plaque instability, making the plaque more prone to rupture [9, 108]. The release of these cytokines also leads to the recruitment of additional immune cells to the plaque, thereby perpetuating the inflammatory cycle and accelerating plaque progression.

In addition to the *NLRP3* inflammasome, other inflammasomes, such as the Absent in Melanoma 2 (*AIM2*) and NLR Family CARD Domain-Containing Protein 4 (*NLRC4*) inflammasomes, are also implicated in atherosclerosis, though their roles are less well understood [109]. Importantly, these inflammasomes can be modulated by various endogenous factors, such as lipids and oxidative stress, both of which are elevated in atherosclerosis. The activation of these inflammasomes within the plaque provides a critical link between lipid metabolism, immune activation, and inflammation in the pathogenesis of atherosclerosis.

The *NLRP3* inflammasome is one of the most well-characterized inflammasomes involved in atherosclerosis, and it is tightly regulated by various cellular components, including *CARD8* [45, 46]. *CARD8*, a negative regulator of *NLRP3*, plays a crucial role in limiting the overactivation of the inflammasome [110]. By binding to *NLRP3*, *CARD8* inhibits its activation and prevents excessive production of IL-1β and other inflammatory mediators [111, 112]. This regulatory function of *CARD8* is important for maintaining a balanced immune response in atherosclerotic plaques, as excessive inflammasome activation can lead to uncontrolled inflammation, plaque destabilization, and the progression of atherosclerosis [51, 53].

The balance between *CARD8* and *NLRP3* activity is critical in controlling the inflammatory environment in atherosclerotic plaques. Increased expression of *CARD8* in macrophages and other immune cells within the plaque may serve as a compensatory mechanism to counteract the harmful effects of excessive inflammasome activation [50]. This highlights the potential of *CARD8* as a therapeutic target for modulating inflammasome activity and reducing the chronic inflammation that accelerates atherosclerotic progression (Table 1).

**Table 1.** **Comparison of CARD8-regulated inflammasome pathways**.

Pathway/Factor	CARD8’s role	Mechanism of action	Impact on atherosclerosis
NLRP3 Inflammasome	CARD8 inhibits NLRP3 inflammasome activation, reducing inflammatory responses	CARD8 interacts with NLRP3 protein, regulating its activation state	Reduces immune cell infiltration into the arterial wall, alleviates plaque formation
IL-1β	CARD8 may modulate IL-1β release, affecting immune responses	Regulates synthesis and secretion of IL-1β through inflammasome pathways	Elevated IL-1β exacerbates atherosclerosis; CARD8’s role may counteract this inflammation
IL-18	CARD8 may modulate IL-18 levels, influencing immune responses	CARD8’s action on NLRP3 inflammasome could affect IL-18 secretion	IL-18 levels correlate with worsening atherosclerosis; CARD8 modulation may reduce this effect
TNF-α	Indirect regulation of TNF-α release	CARD8 modulates NLRP3 inflammasome activation, indirectly affecting TNF-α production	Excessive TNF-α contributes to atherosclerosis progression, and CARD8 may mitigate this

CARD8, Caspase Recruitment Domain Family Member 8; NLRP3, NOD-like Receptor Pyrin Domain-Containing 3; IL, Interleukin; TNF, Tumor Necrosis Factor.

## 4. *CARD8*: An Emerging Key Regulator in Inflammatory Responses

### 4.1 Basic Characteristics of CARD8

*CARD8* is a member of the CARD family of proteins, which includes other well-known inflammasome regulators such as *NLRP3*, ASC, and CASP1 [113]. The *CARD8* gene is located on chromosome 1, and the protein contains a CARD at its N-terminus, which facilitates interactions with other CARD-containing proteins [51]. *CARD8* does not possess caspase-like protease activity but plays a crucial role in regulating the activation of inflammasomes, particularly the *NLRP3* inflammasome [46].

The protein structure of *CARD8* consists of a CARD domain at the N-terminal, followed by a central coiled-coil domain and a C-terminal domain [114]. The CARD domain allows *CARD8* to interact with other CARD-containing proteins, such as *NLRP3* and *ASC*, thereby modulating inflammasome activation [115]. The coiled-coil domain is thought to mediate protein-protein interactions, and the C-terminal domain is involved in the regulation of *CARD8* stability and function [115]. Importantly, the structure of *CARD8* allows it to function as a negative regulator of the *NLRP3* inflammasome, which is crucial in controlling the inflammatory response in atherosclerosis and other inflammatory diseases.

### 4.2 Function of CARD8 in Immune Response

*CARD8* is primarily involved in modulating the innate immune response, particularly through its interaction with the *NLRP3* inflammasome [116]. The *NLRP3* inflammasome is a multi-protein complex that is activated in response to a variety of danger signals, including pathogen-associated molecular patterns (PAMPs) and damage-associated molecular patterns (DAMPs) [117, 118]. When activated, *NLRP3* triggers the activation of caspase-1, which subsequently processes pro-IL-1β and pro-IL-18 into their active forms [119, 120]. These cytokines are released into the extracellular space, where they drive inflammation and immune cell recruitment to sites of injury or infection. *CARD8* serves as a negative regulator of *NLRP3* by interacting with the CARD domain of *NLRP3* and preventing its activation [46]. In this way, *CARD8* helps to suppress the excessive release of IL-1β and IL-18, which are potent pro-inflammatory cytokines involved in chronic inflammation, tissue damage, and atherosclerosis progression. By inhibiting *NLRP3* activation, *CARD8* helps to maintain a balanced immune response, ensuring that the inflammatory process does not become dysregulated and lead to excessive tissue damage or plaque instability. In addition to its role in regulating inflammasomes, *CARD8* has been shown to interact with other signaling pathways, including the Nuclear Factor kappa-light-chain-enhancer of activated B cells (NF-κB) pathway, which is involved in the production of pro-inflammatory cytokines. Through these interactions, *CARD8* further contributes to the resolution of inflammation and the prevention of chronic inflammatory diseases such as atherosclerosis.

### 4.3 Expression and Regulation of CARD8: Key to Understanding its Function

*CARD8* is expressed in a variety of immune cells, including macrophages, dendritic cells, and T lymphocytes [44, 51, 121]. The expression of *CARD8* is tightly regulated in response to inflammatory stimuli, and its levels can be upregulated in response to danger signals such as ox-LDL and other DAMPs [122]. Studies have shown that the expression of *CARD8* is particularly high in macrophages, where it plays a critical role in controlling the inflammatory response within atherosclerotic plaques [123]. In these cells, *CARD8* interacts with the *NLRP3* inflammasome to limit the activation of IL-1β and IL-18, thereby reducing the inflammatory burden in the plaque [124]. In addition to macrophages, *CARD8* expression has been detected in other immune cells involved in atherosclerosis, such as dendritic cells and T lymphocytes [43]. Dendritic cells are important antigen-presenting cells that activate T lymphocytes and regulate adaptive immune responses in atherosclerotic lesions [123]. The expression of *CARD8* in dendritic cells suggests that it may play a role in modulating the adaptive immune response in atherosclerosis, potentially influencing the balance between pro-inflammatory and regulatory T cells [43].

The regulation of *CARD8* expression is also influenced by various transcription factors, including NF-κB and Activator Protein (AP)-1, which are activated in response to inflammatory signals [125]. These transcription factors promote the expression of *CARD8* in immune cells, particularly during times of inflammation [125]. Moreover, *CARD8* itself can be regulated by post-translational modifications, such as phosphorylation, which can alter its stability and activity [126]. The precise mechanisms that regulate *CARD8* expression and activity in atherosclerosis remain an active area of research [43].

### 4.4 CARD8 in Atherosclerosis: Implications for Disease Progression

*CARD8* plays a critical role in modulating the immune response in atherosclerosis by regulating the activation of the *NLRP3* inflammasome and other inflammatory pathways. In atherosclerotic plaques, the interaction between *CARD8* and *NLRP3* helps to suppress excessive inflammasome activation, thereby reducing the release of IL-1β and IL-18 [6]. This, in turn, helps to limit the recruitment and activation of immune cells, such as macrophages and T lymphocytes, which are responsible for driving the chronic inflammation in the plaque [127].

In macrophages, *CARD8* limits the production of pro-inflammatory cytokines, which helps to stabilize the plaque and prevent plaque rupture [43]. In addition, *CARD8* modulates the activation of T lymphocytes, particularly Tregs, which play a key role in suppressing inflammation and promoting plaque stability [128]. By influencing both the innate and adaptive immune responses, *CARD8* contributes to the resolution of inflammation in atherosclerosis and plays a protective role in maintaining plaque stability [113]. Furthermore, *CARD8*’s regulatory function extends beyond inflammasome inhibition [113]. While *CARD8* predominantly acts as a negative regulator of *NLRP3* inflammasome activation in atherosclerosis by binding to *NLRP3* and inhibiting its oligomerization with ASC, thereby limiting IL-1β and IL-18 release to prevent excessive inflammation in plaques [113], emerging evidence reveals a context-dependent duality where *CARD8* can promote pro-inflammatory responses under specific conditions, such as in macrophage activation and foam cell formation. This functional switch is triggered by molecular cues like pathogen-derived proteases or dysregulated proteostasis, where *CARD8* undergoes autoproteolytic processing at its Function to Find Domain (FIIND) domain, releasing a bioactive C-terminal fragment (UPA-CARD) that directly recruits and activates caspase-1, independent of ASC [50, 115]. For instance, in lipid-laden environments mimicking foam cell formation, the T60 isoform of *CARD8*—prevalent in immune cells—senses Human Immunodeficiency Virus (HIV)-1 protease activity, leading to N-terminal cleavage, proteasome-mediated degradation of the inhibitory fragment, and subsequent *CARD8* inflammasome assembly, which induces pyroptosis and IL-1β secretion in macrophages [129]. This pro-inflammatory activation may exacerbate plaque instability by amplifying cytokine-driven immune cell recruitment and necrotic core expansion, particularly in the presence of viral co-infections or oxidative stress that unfolds *CARD8*’s disordered N-terminal region, enhancing its susceptibility to degradation and sensor function [114]. Genetic variants, such as rs2043211 (C10X), further modulate this duality; the minor allele disrupts the T48 isoform’s inhibitory binding to *NLRP3*, potentially shifting toward pro-inflammatory *CARD8* activation in heterozygous individuals, as observed in inflammatory diseases with atherosclerotic overlap [130]. These mechanisms underscore *CARD8*’s isoform- and stimulus-specific roles, highlighting the need for targeted therapies that preserve its anti-inflammatory function while mitigating pro-inflammatory activation in advanced plaques. *CARD8* has been implicated in regulating the migration and activation of immune cells, particularly macrophages and T lymphocytes, within the atherosclerotic plaque [43]. By controlling immune cell function and limiting excessive inflammation, *CARD8* helps to reduce plaque progression and the risk of thrombosis [111].

A study by Paramel Varghese *et al*. [106] examined *CARD8* mRNA expression in atherosclerotic vascular tissue and compared it to transplant donor arterial tissue. They found that *CARD8* mRNA was highly expressed in atherosclerotic plaques compared to the expression in transplant donor vessels [51]. Another research used immunohistochemistry to examine *CARD8* expression in non-atherosclerotic arteries and carotid lesions. In non-atherosclerotic vessels, *CARD8* expression was primarily detected in endothelial cells and smooth muscle cells in the tunica media. In atherosclerotic carotid lesions, *CARD8* was detected in the endothelial layer, smooth muscle cells, and CD68^+^ macrophages, suggesting that immune cells, along with vascular cells, contribute to the increased expression of *CARD8* in human atherosclerotic lesions [43]. These studies indicate that *CARD8* expression indeed varies between normal and atherosclerotic tissues. However, more research is needed to comprehensively understand its expression differences across diverse atherosclerosis models, such as those induced by different risk factors (e.g., high- fat diet - induced, oxidized LDL - induced).

## 5. *CARD8* in Atherosclerosis: From Bench to Bedside

### 5.1 The Role of CARD8 in Atherosclerosis Progression

Atherosclerosis is a chronic inflammatory disease in which immune cell activation, lipid deposition, and extracellular matrix remodeling contribute to the thickening of the arterial walls and the formation of plaques [131]. The inflammatory response plays a pivotal role in all stages of atherosclerosis, from the initiation of endothelial injury to the destabilization and rupture of advanced plaques [131]. As a negative regulator of the *NLRP3* inflammasome, *CARD8* has emerged as a key player in controlling the balance between inflammation and tissue repair in atherosclerosis [113].

The expression of *CARD8* in atherosclerotic lesions has been shown to influence plaque stability and progression [43]. In animal models, the overexpression of *CARD8* in macrophages leads to the suppression of IL-1β and IL-18 production, reducing the inflammatory burden within the plaque [113]. This results in the stabilization of the plaque, as lower levels of inflammatory cytokines reduce immune cell recruitment and smooth muscle cell proliferation [132]. Conversely, a lack of *CARD8* or its inhibition exacerbates the inflammatory response, promoting plaque progression and destabilization, which increases the risk of rupture and subsequent cardiovascular events [43]. Several studies have demonstrated that *CARD8* interacts with other inflammatory pathways in atherosclerosis [43]. For example, *CARD8* can modulate the NF-κB signaling pathway, which is activated in response to inflammatory stimuli [113]. By inhibiting excessive NF-κB activation, *CARD8* helps to limit the production of pro-inflammatory cytokines such as TNF-α and IL-6, further reducing inflammation within the plaque [43]. In addition to its direct modulation of NF-κB, *CARD8* engages in potential crosstalk with other key inflammatory pathways, such as mitogen-activated protein kinase (MAPK) and Janus kinase-Signal Transducer and Activator of Transcription (JAK-STAT), to regulate cytokine production and immune cell function in atherosclerosis. *CARD8* inhibits NF-κB activation via interaction with the IκB kinase complex (IκB kinase γ subunit (IKKγ)/NF-κB essential modulator (NEMO)), suppressing downstream cytokine expression (e.g., TNF-α, IL-6) in endothelial cells and macrophages, which may indirectly influence JAK-STAT signaling given IL-6’s role in activating JAK/STAT3 to induce monocyte chemoattractant protein-1 (MCP-1) in vascular endothelial cells [43]. Although direct interactions with MAPK are not well-established, *CARD8*’s regulation of inflammasome activity could intersect with MAPK pathways, as *NLRP3* inhibition by *CARD8* limits p38 MAPK-mediated inflammatory responses in immune cells, potentially reducing cytokine-driven plaque progression [113]. In atherosclerotic lesions, this network integration helps maintain immune homeostasis by dampening macrophage activation and T cell polarization, with *CARD8* overexpression in *ApoE*^-⁣/-^ models attenuating IL-1β and IL-18 release, which otherwise amplifies NF-κB-JAK-STAT crosstalk to exacerbate foam cell formation and plaque instability [43]. These mechanisms underscore *CARD8*’s multifaceted role in orchestrating inflammatory signaling, though further studies are needed to elucidate direct crosstalk with MAPK and JAK-STAT in human plaques. This highlights *CARD8*’s multifaceted role in controlling inflammation and plaque stability, making it a potential therapeutic target in atherosclerosis.

### 5.2 Expression and Functional Data of CARD8 in Atherosclerosis

Experimental studies have provided valuable insights into the role of *CARD8* in atherosclerosis. In *ApoE*^-⁣/-^ mice, a commonly used animal model of atherosclerosis, *CARD8* expression is upregulated in the aortic tissues, particularly in macrophages and other immune cells within atherosclerotic plaques [43]. Studies have shown that the deletion of *CARD8* in these mice leads to increased levels of pro-inflammatory cytokines such as IL-1β and IL-18, along with greater immune cell infiltration into the plaque [112]. These findings support the notion that *CARD8* acts as a negative regulator of inflammation and plaque development.

In contrast, the overexpression of *CARD8* in macrophages has been shown to attenuate the inflammatory response in these animal models, leading to a reduction in plaque size and improved plaque stability [43]. This protective effect is attributed to *CARD8*’s ability to inhibit *NLRP3* inflammasome activation, preventing the excessive release of IL-1β and IL-18, which are known to drive inflammation in atherosclerotic lesions [133]. The overexpression of *CARD8* also leads to a decrease in the number of foam cells in the plaque, suggesting that *CARD8* may influence lipid metabolism and foam cell formation [43]. Immunohistochemical analysis of human atherosclerotic plaques reveals that *CARD8* is expressed in macrophages and smooth muscle cells within the plaque [43]. Notably, the expression of *CARD8* is inversely correlated with the levels of IL-1β in the plaque, suggesting that *CARD8* acts to dampen the inflammatory response in human atherosclerosis as well [43]. Furthermore, elevated *CARD8* expression has been associated with a more stable plaque phenotype, characterized by a thicker fibrous cap and fewer signs of inflammation (Table 2, Ref. [43, 80, 113, 133, 134]) [43, 134]. Differential expression of *CARD8* across atherosclerosis models highlights its context-specific roles in regulating inflammation and plaque stability. In *ApoE*^-⁣/-^ mice, *CARD8* is significantly upregulated in aortic macrophages and smooth muscle cells within atherosclerotic lesions, correlating with reduced IL-1β and IL-18 levels, which mitigates plaque progression and enhances stability [43]. Conversely, in Low-Density Lipoprotein Receptor (*LDLR*)^-⁣/-^ mice, *CARD8* expression is generally lower under hyperlipidemic conditions, potentially due to heightened oxidative stress and lipid accumulation, leading to increased *NLRP3* inflammasome activity and more severe plaque inflammation [79]. Human atherosclerotic plaques exhibit heterogeneous *CARD8* expression, predominantly in macrophages and foam cells, with higher levels in stable plaques compared to unstable ones, inversely correlating with IL-1β expression [43]. A 2024 study further revealed that the *CARD8* rs2043211 polymorphism modulates expression in human monocytes, with the minor allele reducing *CARD8* mRNA levels in males, potentially exacerbating inflammatory responses in early atherosclerosis [135]. *In vitro*, human endothelial cells exposed to ox-LDL show induced *CARD8* expression, which suppresses adhesion molecule expression (e.g., Intercellular Adhesion Molecule (ICAM)-1) to limit monocyte recruitment, contrasting with the macrophage-centric anti-inflammatory role in animal models [43]. These variations suggest that *CARD8* expression is influenced by model-specific factors, such as lipid metabolism, genetic background, and inflammatory stimuli, underscoring the need for tailored therapeutic strategies targeting *CARD8* in atherosclerosis [113].

**Table 2.** **Comparative analysis of key outcomes in *ApoE*^-⁣/-^ mouse models of atherosclerosis with *CARD8* deletion versus overexpression**.

Experimental manipulation	Plaque size	Cytokine levels (IL-1β, IL-18)	Foam cell counts	Other outcomes	Reference
*CARD8* deletion	Increased plaque area in aortic tissues	Elevated IL-1β and IL-18 production	Increased foam cell formation and macrophage infiltration	Enhanced immune cell recruitment, reduced plaque stability	[133]
*CARD8* overexpression	Reduced plaque size	Decreased IL-1β and IL-18 levels	Decreased foam cell numbers	Improved plaque stability, attenuated inflammatory response	[43, 113]
*NLRP3* deletion	No significant change in plaque progression	Reduced IL-1β release	No major alteration in foam cells	Independent of *NLRP3* in some *ApoE*^-⁣/-^ contexts, minimal impact on hypercholesterolemia	[134]
*NLRP3* inhibition	Reduced atherosclerotic lesion area	Suppressed IL-1β and IL-18	Decreased foam cell accumulation	Stabilized plaques, reduced calcification	[80]

### 5.3 Immune Cells in Atherosclerosis: CARD8’s Impact on Macrophages, Monocytes, and T Cells

The role of *CARD8* in immune cells, particularly macrophages, has been extensively studied in the context of atherosclerosis. Macrophages are the primary immune cells involved in the formation and progression of atherosclerotic plaques. Upon recruitment to the site of injury, monocytes differentiate into macrophages, where they play a key role in phagocytosing ox-LDL and forming foam cells [136]. Foam cells contribute to plaque formation and progression by secreting pro-inflammatory cytokines that perpetuate the inflammatory cycle [137].

*CARD8* has been shown to regulate the inflammatory response in macrophages by inhibiting *NLRP3* inflammasome activation [113]. In macrophages from *CARD8*-deficient mice, there is a marked increase in IL-1β production and foam cell formation, leading to more severe plaque progression [138]. This suggests that *CARD8* plays a crucial role in maintaining macrophage function and preventing excessive inflammation in the plaque [139]. Conversely, macrophages overexpressing *CARD8* exhibit reduced levels of IL-1β and IL-18, along with a decrease in foam cell formation, leading to a more stable plaque [113].

*CARD8* is also expressed in other immune cells, including monocytes and T lymphocytes, which are involved in the adaptive immune response in atherosclerosis. In monocytes, *CARD8* regulates the activation of the *NLRP3* inflammasome and helps to control the production of IL-1β, which is essential for monocyte migration and differentiation into macrophages [113]. In T cells, particularly T helper cells, *CARD8* may influence the balance between pro-inflammatory Th1 cells and anti-inflammatory Tregs, which has implications for plaque stability and immune regulation [42] (Table 3). Beyond its role in monocytes and macrophages, *CARD8* significantly influences dendritic cell (DC) and T lymphocyte functions, modulating adaptive immune responses critical to atherosclerotic plaque dynamics. In DCs, *CARD8* is expressed at moderate levels and regulates antigen presentation by limiting *NLRP3* inflammasome activation, which reduces IL-1β-driven DC maturation and subsequent T cell priming [43]. This dampening effect promotes tolerogenic DC phenotypes, enhancing the induction of Tregs over pro-inflammatory Th1 cells in atherosclerotic lesions, as evidenced by reduced IFN-γ and increased IL-10 expression in *CARD8*-overexpressing DC-T cell co-cultures [113]. In T lymphocytes, particularly CD4^+^ T cells, *CARD8* modulates polarization by inhibiting caspase-1-mediated pyroptosis, preserving Treg survival and function, which is critical for maintaining immune homeostasis and plaque stability [42]. A 2023 study demonstrated that *CARD8* expression in T cells from human atherosclerotic plaques correlates with higher Treg/Th1 ratios, suggesting a protective role against excessive Th1-driven inflammation [87]. Furthermore, *CARD8*’s interaction with NF-κB pathways in DCs suppresses pro-inflammatory cytokine production (e.g., IL-12), which otherwise skews T cell differentiation toward Th1 cells, exacerbating plaque progression [43]. These findings highlight *CARD8*’s role in DC-T cell crosstalk, where it fosters an anti-inflammatory microenvironment by balancing antigen presentation and T cell polarization, offering potential therapeutic avenues to enhance plaque stability through targeted modulation of adaptive immunity.

**Table 3.** **Comparative function of card8 in different immune cells**.

Immune cell type	CARD8 expression level	Function of CARD8 in immune cells	Impact on atherosclerosis
Macrophages	High	Regulates NLRP3 inflammasome activation, modulates inflammatory response	Promotes progression of atherosclerosis by enhancing local inflammation
Monocytes	Moderate	Involved in innate immune responses, induces cytokine release	Contributes to immune cell infiltration in the arterial wall, promoting plaque formation
T cells	Low	Modulates T cell activation, influences Th1/Th2 balance	Indirect effect on atherosclerosis via modulation of immune microenvironment
Endothelial cells	Very low or none	Not directly expressed, but may mediate through immune cells	Indirect influence on atherosclerosis through immune cell activation

### 5.4 CARD8 in Lipid Deposition and Plaque Inflammation

Beyond its role in immune regulation, *CARD8* may also influence lipid deposition within the atherosclerotic plaque. Foam cell formation is a key event in atherosclerosis, and macrophages play a central role in the uptake of ox-LDL, which contributes to lipid accumulation in the plaque [140]. Recent studies suggest that *CARD8* may modulate lipid metabolism in macrophages by regulating the expression of genes involved in lipid uptake and efflux [43]. In particular, *CARD8* has been shown to inhibit the expression of pro-inflammatory lipid receptors, such as scavenger receptors, which are responsible for the uptake of ox-LDL into macrophages [140]. In addition, *CARD8* may influence plaque inflammation through its effects on smooth muscle cells. Smooth muscle cells contribute to the formation of the fibrous cap in advanced plaques, and their proliferation is driven by inflammatory signals [141]. By suppressing the activation of pro-inflammatory pathways in smooth muscle cells, *CARD8* may help maintain plaque stability and prevent plaque rupture [141].

### 5.5 Clinical Insights: Evidence for CARD8 as a Diagnostic and Therapeutic Target

Clinical studies investigating the role of *CARD8* in human atherosclerosis have provided further evidence of its involvement in plaque stability. Immunohistochemical analysis of human atherosclerotic plaques reveals that *CARD8* expression is significantly higher in stable plaques compared to unstable plaques [43]. Moreover, higher levels of *CARD8* in plaque macrophages are associated with lower levels of IL-1β and reduced immune cell infiltration [43]. These clinical observations are supported by immunohistochemical (IHC) and gene expression analyses from well-characterized patient cohorts. In the Biobank of Karolinska Endarterectomies (BiKE) study, carotid atherosclerotic plaques were obtained from 126 patients undergoing endarterectomy for ischemic cerebrovascular disease (advanced symptomatic atherosclerosis), with non-atherosclerotic control vessels from transplant donors [43]. Comorbidities in this cohort included hypertension, diabetes, and hyperlipidemia, common in advanced atherosclerosis, though specific prevalence rates were not stratified for *CARD8* analysis. *CARD8* protein expression was assessed via IHC on formalin-fixed, paraffin-embedded sections (4 µm) using anti-*CARD8* antibodies, visualized with 3,3^′^-diaminobenzidine (DAB), and semi-quantitatively scored based on staining intensity and cellular localization in macrophages and smooth muscle cells, revealing higher expression in stable plaques with thicker fibrous caps. Complementary microarray analysis (Affymetrix HG-U133 plus 2.0) on RNA from 106 BiKE plaques (normalized via robust multi-array average on log2 scale) showed *CARD8* mRNA upregulation in plaques versus controls, with inverse correlations to IL-1β levels after Benjamini-Hochberg false discovery rate adjustment [51]. These methods underscore *CARD8*’s association with reduced inflammation in stable plaques, though larger cohorts with quantitative digital pathology scoring could further validate translational applicability. These findings suggest that *CARD8* may serve as a marker of plaque stability and may help predict the risk of plaque rupture in patients with atherosclerosis.

Despite promising associations with plaque stability, translating *CARD8* as a biomarker into clinical practice faces several hurdles, including assay development for detection in blood versus plaque tissue and establishing correlations with clinical endpoints like major adverse cardiovascular events (MACE). *CARD8* is primarily an intracellular protein, complicating its detection in circulation; current methods rely on IHC analysis of plaque tissue or mRNA quantification via microarray in cohorts like the BiKE study, where *CARD8* expression inversely correlates with IL-1β but lacks direct blood-based assays such as ELISA due to low solubility and absence of secreted forms [43]. Genetic polymorphisms, such as rs2043211 (C10X variant), serve as potential proxies for *CARD8* function, with the minor allele associated with reduced *CARD8* activity and increased inflammatory markers in healthy individuals, but their predictive value for atherosclerosis progression remains limited without large-scale validation [135]. Correlation with MACE is underexplored; in abdominal aortic aneurysms, *CARD8* variants interact with *NLRP3* to influence disease risk, but no direct links to cardiovascular events like rupture or infarction have been established, highlighting the need for prospective studies integrating *CARD8* genetics with imaging or circulating inflammation markers [47]. Additional challenges include assay sensitivity for low-abundance proteins in blood, variability due to comorbidities (e.g., hypertension, diabetes), and the requirement for standardized quantitation methods to enable routine clinical use [46, 113]. Overcoming these barriers could position *CARD8* as a viable biomarker for risk stratification in atherosclerosis.

The therapeutic potential of *CARD8* as a target for atherosclerosis treatment is supported by its ability to modulate inflammation and immune cell function within plaques [43]. By enhancing *CARD8* expression or activity, it may be possible to reduce the inflammatory burden in atherosclerosis, prevent plaque destabilization, and improve patient outcomes [113]. However, further studies are needed to determine the exact mechanisms by which *CARD8* influences plaque biology and to evaluate its potential as a therapeutic target in clinical settings [39].

## 6. Therapeutic Potential of Targeting *CARD8* in Atherosclerosis

### 6.1 Targeting CARD8: A Promising Strategy for Atherosclerosis Therapy

As a negative regulator of the *NLRP3* inflammasome, *CARD8* has emerged as a promising therapeutic target for atherosclerosis, a disease primarily driven by inflammation. The idea of modulating *CARD8* expression or function to control the inflammatory response in atherosclerotic lesions offers a novel approach to treatment [113]. Given that atherosclerosis is characterized by chronic inflammation, which leads to plaque instability, targeted therapies aimed at restoring the regulatory function of *CARD8* could potentially halt or even reverse disease progression [142].

Preclinical studies have highlighted the potential of *CARD8* as a therapeutic target. By inhibiting excessive activation of the *NLRP3* inflammasome, *CARD8* can reduce the production of IL-1β and IL-18, which are critical cytokines driving inflammation and atherosclerotic plaque formation. The ability to restore *CARD8* function in macrophages and other immune cells could lead to the stabilization of plaques, reducing the likelihood of plaque rupture and the risk of cardiovascular events, such as myocardial infarction and stroke [111].

There are several strategies for targeting *CARD8* in the context of atherosclerosis treatment. One promising approach is the use of small molecules that can enhance *CARD8* expression or activate its function [137]. Alternatively, the development of monoclonal antibodies that mimic *CARD8*’s regulatory effects on the *NLRP3* inflammasome could provide a more targeted and specific therapeutic intervention [111].

### 6.2 Current Approaches and Therapeutic Strategies for Targeting CARD8

Although direct therapeutic strategies targeting *CARD8* are still in the early stages of development, several approaches have been explored in related inflammatory diseases, which could provide insight into potential therapies for atherosclerosis [143].

One potential strategy involves the use of small molecules that modulate the activity of the inflammasome. For example, inhibitors of the *NLRP3* inflammasome, such as *MCC950*, have shown promise in reducing systemic inflammation and preventing disease progression in models of atherosclerosis [144]. These inhibitors could work synergistically with *CARD8*, either by increasing its activity or by restoring its function in cases of *CARD8* deficiency [145]. As the role of *CARD8* in inflammasome regulation becomes clearer, drugs that specifically target *CARD8*’s interaction with *NLRP3* could be developed to provide more precise modulation of the immune response [146].

Another potential approach involves gene therapy to enhance the expression of *CARD8* in atherosclerotic plaques. Using viral vectors or mRNA-based delivery systems, the therapeutic delivery of *CARD8* could help restore its normal function in immune cells, particularly macrophages, where its effect on inflammasome regulation is most significant [147]. Preclinical studies in animal models of atherosclerosis have demonstrated the potential for gene therapy to reduce plaque size and improve plaque stability, with *CARD8* playing a central role in this effect [111].

Additionally, monoclonal antibodies or recombinant proteins that mimic *CARD8*’s anti-inflammatory effects could offer a more targeted approach to modulating the inflammasome in atherosclerosis [111]. These therapies would be designed to activate *CARD8*’s role in inhibiting *NLRP3* activation, thereby reducing IL-1β and IL-18 levels and limiting the inflammatory processes that drive atherosclerosis [148] (Table 4). Preliminary preclinical data on candidate molecules and delivery platforms provide insights into targeting *CARD8*’s regulatory function in atherosclerosis. For small molecules, MCC950, a selective *NLRP3* inhibitor, synergizes with *CARD8*’s inhibitory role by blocking *NLRP3* activation at nanomolar concentrations, reducing IL-1β release in human monocyte-derived macrophages [149]. In *ApoE*^-⁣/-^ mouse models of atherosclerosis, MCC950 administered intraperitoneally hindered plaque development, attenuating macrophage pyroptosis and inflammation, with a 30–40% reduction in aortic lesion area and lowered serum IL-1β levels, demonstrating dose-dependent efficacy without significant toxicity [150]. Monoclonal antibodies targeting downstream pathways, such as canakinumab (anti-IL-1β), mimic *CARD8*’s anti-inflammatory effects; in preclinical rabbit models of atherosclerosis, subcutaneous dosing stabilized plaques by reducing IL-1β-driven inflammation, with a 25% decrease in macrophage infiltration and improved fibrous cap thickness [151]. These examples highlight progress in *CARD8*-related therapeutics, though direct *CARD8* agonists remain under development, emphasizing the need for *CARD8*-specific lead optimization.

**Table 4.** **Comparison of CARD8-targeted therapeutic strategies**.

Therapeutic strategy	Mechanism of action	Preclinical results	Challenges in preclinical research
Small molecule inhibitors	Inhibit CARD8 expression or function, reduce NLRP3 inflammasome activation	Small molecule inhibitors effectively reduce CARD8 expression and slow atherosclerosis progression in animal models	Selectivity, off-target effects, and potential toxicity require further validation
Antibody therapy	Antibodies bind to CARD8, inhibiting its function, blocking inflammasome activation	In animal models, anti-CARD8 antibodies reduced atherosclerotic plaque formation	Antibody half-life, stability, and immune tolerance remain major challenges
Gene editing	Knock out CARD8 gene, completely abolishing its function	CRISPR-Cas9 effectively deleted the CARD8 gene in animal models, reducing atherosclerosis symptoms	Off-target effects and long-term safety concerns need further evaluation
Vaccine therapy	Immunization to activate or suppress CARD8-specific functions	Immunization may activate specific immune responses, though efficacy in atherosclerosis is under further investigation	Immunogenicity and safety of the vaccine need more clinical trials

### 6.3 Preclinical Insights: Experimental Models of CARD8 Inhibition

A variety of preclinical studies have investigated the role of *CARD8* in atherosclerosis and its potential as a therapeutic target [152]. In animal models, the inhibition or deletion of *CARD8* exacerbates plaque formation and inflammation, leading to unstable plaques that are prone to rupture [153]. These findings suggest that restoring *CARD8* function could stabilize plaques and reduce the incidence of cardiovascular events. Gene knockout studies in *ApoE*^-⁣/-^ mice have demonstrated that *CARD8* deficiency accelerates the development of atherosclerotic lesions and increases the inflammatory cytokine production in the plaques [148]. Restoring *CARD8* expression in these animals significantly reduces plaque size and stabilizes the lesions. Additionally, the modulation of *CARD8* activity through gene therapy or small molecules has been shown to reduce the inflammatory response, improving plaque stability and reducing the risk of plaque rupture [111].

*In vitro* studies using macrophage and endothelial cell cultures have further confirmed the role of *CARD8* in regulating the inflammatory response in atherosclerosis. For instance, activating *CARD8* in macrophages has been shown to inhibit *NLRP3* inflammasome activation, reduce IL-1β secretion, and prevent foam cell formation [111]. These cellular models have provided valuable insights into the mechanisms by which *CARD8* modulates inflammation and plaque progression, and they will be essential for developing targeted therapies [152].

### 6.4 Clinical Challenges and Future Considerations

While the therapeutic potential of *CARD8* in atherosclerosis is promising, several challenges must be addressed before it can be translated into clinical practice [154]. One major challenge is the need for specific and safe targeting of *CARD8*. Given the complexity of the immune system and the fact that *CARD8* is involved in regulating multiple immune responses, any therapeutic intervention must be carefully designed to avoid unintended effects on other aspects of immune function [143].

Another challenge lies in the delivery of *CARD8*-targeting therapies to the atherosclerotic lesions. Efficient and targeted delivery of therapeutic agents to specific cells, such as macrophages in plaques, remains a significant hurdle in the field of cardiovascular disease treatment [155]. Advances in nanotechnology and targeted drug delivery systems may help overcome these obstacles, but further research is needed to optimize these approaches [156].

Finally, while preclinical studies have demonstrated the potential benefits of *CARD8* modulation, clinical trials in humans are required to assess the safety and efficacy of such therapies [153]. The success of clinical trials will depend on the ability to identify suitable biomarkers for *CARD8* activity and monitor the effects of treatment on plaque progression and stability [157]. Additionally, the potential for off-target effects and long-term safety must be thoroughly evaluated before *CARD8*-targeting therapies can be introduced into clinical practice [158].

Long-term safety challenges for *CARD8*-targeted therapies, which primarily inhibit *NLRP3* inflammasome activation, include unintended immunosuppression, off-target effects on other inflammasomes such as AIM2 and NLRC4, and compromised host defense mechanisms, potentially increasing infection risk in chronic inflammatory conditions like atherosclerosis [159]. Unintended immunosuppression arises from *NLRP3*’s role in innate immunity; chronic inhibition may impair pathogen clearance, as evidenced by increased susceptibility to bacterial and viral infections in preclinical models treated with *NLRP3* inhibitors like MCC950, where long-term dosing reduced IL-1β but elevated infection rates in mice challenged with pathogens [160]. Off-target effects on AIM2 and NLRC4 are a concern, as *CARD8* mutations marginally impact AIM2 activation without affecting NLRC4 or pyrin, potentially leading to dysregulated DNA-sensing (AIM2) or bacterial defense (NLRC4) in immune cells, exacerbating opportunistic infections or autoimmune flares. Impacts on host defense are highlighted in studies showing *NLRP3*’s beneficial early-stage role in infection recognition; selective inhibitors preserve some immune functions but risk broader immunosuppression in vulnerable populations, such as those with comorbidities, necessitating monitoring for MACE and infections in trials [161]. These risks underscore the importance of developing *CARD8*-specific agents with minimal off-target activity to ensure clinical feasibility.

### 6.5 Synergistic Approaches: Combining CARD8 Inhibition With Other Therapeutic Modalities

Given the complex pathophysiology of atherosclerosis, combination therapies targeting *CARD8* along with other inflammatory pathways could offer a more effective treatment strategy [162]. For example, combining *CARD8* modulation with existing anti-inflammatory therapies, such as IL-1β inhibitors, may provide synergistic effects in reducing systemic inflammation and stabilizing plaques [163]. Moreover, combining *CARD8*-targeted therapies with lipid-lowering agents, such as statins, could further enhance the therapeutic benefits by addressing both the inflammatory and lipid components of atherosclerosis [164]. The combination of *CARD8*-targeting strategies with lifestyle interventions, such as diet and exercise, could also play a role in managing atherosclerosis and reducing the need for invasive procedures [165]. Further research into the mechanisms of action of *CARD8* in atherosclerosis will help define the best strategies for combination therapies and improve patient outcomes [166].

## 7. Conclusion and Future Directions

Atherosclerosis remains a leading cause of cardiovascular morbidity and mortality worldwide, and despite significant advancements in therapeutic strategies, the disease continues to pose a major public health challenge. Chronic inflammation is a hallmark of atherosclerosis, and targeting key regulatory pathways involved in immune responses holds considerable promise for novel therapeutic interventions. Among the various immune modulators, *CARD8* has emerged as a critical player in regulating the *NLRP3* inflammasome, a central driver of inflammation in atherosclerosis.

**Fig. 2.**
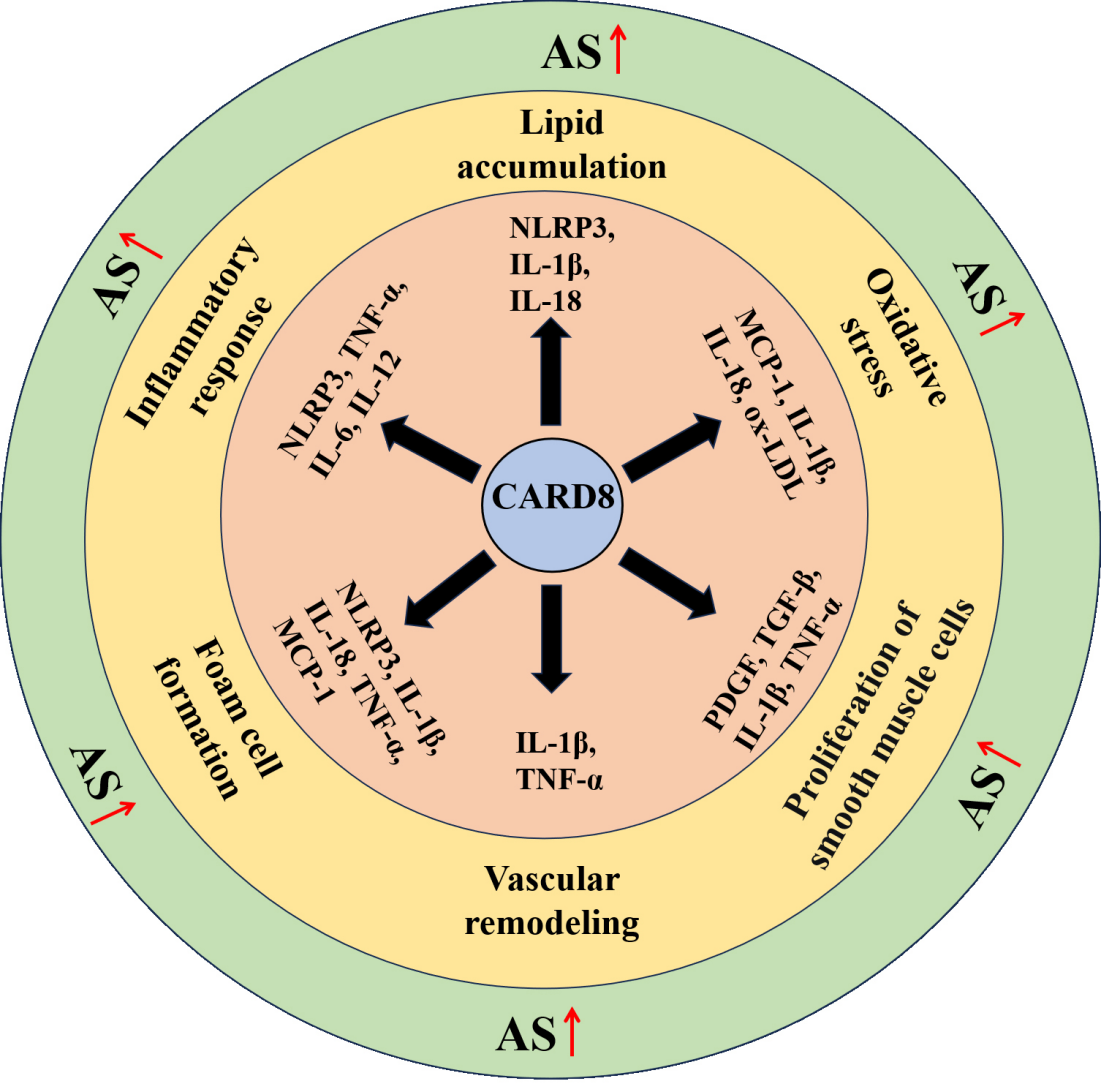
***CARD8*-mediated regulation and the major cardiometabolic risk factors of atherosclerosis**. Arrows in red: promote; AS, atherosclerosis; MCP-1, monocyte chemoattractant protein-1; ox-LDL, oxidized low-density lipoprotein; PDGF, Platelet-Derived Growth Factor; TGF, Transforming Growth Factor.

Through its inhibitory effect on *NLRP3* activation, *CARD8* regulates the production of key pro-inflammatory cytokines, such as IL-1β and IL-18, which contribute to plaque formation, instability, and rupture. Furthermore, emerging evidence suggests that *CARD8* plays a key role in immune cell function, particularly in macrophages, where it helps modulate the inflammatory microenvironment within atherosclerotic plaques. These findings highlight *CARD8*’s potential as a therapeutic target for atherosclerosis, as its modulation could help stabilize plaques, reduce inflammation, and potentially prevent adverse cardiovascular events (Fig. 2).

Despite the promising preclinical data, several challenges remain in translating *CARD8*-targeted therapies into clinical practice. A comprehensive understanding of the molecular mechanisms by which *CARD8* influences immune cell responses, along with the identification of reliable biomarkers, is essential for the development of targeted therapies. Additionally, overcoming obstacles related to the delivery of *CARD8*-modulating agents to atherosclerotic lesions and ensuring their safety and efficacy in long-term clinical trials are critical steps toward the successful clinical application of *CARD8*-targeted therapies.

Looking ahead, future research should focus on addressing these challenges by expanding our understanding of the role of *CARD8* in immune regulation across different stages of atherosclerosis and in various immune cell populations. Moreover, the development of combination therapies that target *CARD8* alongside other inflammatory pathways, such as IL-1β, may provide more effective treatment options for patients with atherosclerosis. Personalized medicine approaches, guided by genetic and immunological profiles, will be essential in optimizing therapeutic strategies for individual patients. In conclusion, *CARD8* holds significant promise as a novel therapeutic target in atherosclerosis. Further research is needed to fully elucidate its molecular mechanisms and to validate its potential in clinical settings. The translation of *CARD8*-based therapies from bench to bedside could lead to more effective treatments for atherosclerosis, ultimately improving patient outcomes and reducing the burden of cardiovascular disease.

Despite the promising role of *CARD8* in atherosclerosis and its potential as a therapeutic target, several important research gaps remain. First, the precise molecular mechanisms by which *CARD8* regulates the *NLRP3* inflammasome and how this influences atherosclerosis progression are not fully understood. While *CARD8* has been established as an inhibitor of *NLRP3* inflammasome activation, its detailed role in the modulation of other immune signaling pathways, such as those involved in macrophage polarization or endothelial dysfunction, requires further investigation. Understanding the broader context in which *CARD8* functions could unveil additional therapeutic opportunities, especially in the context of other cardiovascular diseases that also involve chronic inflammation. Second, the role of *CARD8* in different types of immune cells in the context of atherosclerosis needs to be more thoroughly explored. Although *CARD8* has been predominantly studied in macrophages, its role in other immune cells, such as dendritic cells, T cells, and smooth muscle cells, is less well understood. These cells contribute to various stages of plaque development and stability, and their response to *CARD8* modulation could have significant therapeutic implications. Another major challenge is the heterogeneity of atherosclerosis itself. Atherosclerotic plaques are highly variable, with different stages of progression and varying degrees of inflammation, lipid accumulation, and vascular remodeling. The development of biomarkers to identify patients at different stages of disease, and the role of *CARD8* in these stages, would be crucial for determining the optimal timing and strategy for therapeutic intervention. Moreover, the effects of *CARD8* modulation on plaque stability, rupture, and cardiovascular events need to be clarified through long-term clinical trials.

Future research should prioritize specific gaps to advance *CARD8*’s therapeutic potential in atherosclerosis. First, elucidating *CARD8*’s role in early versus advanced atherosclerotic lesions is critical, as its expression is elevated in stable plaques with thicker fibrous caps but less clear in early lipid-driven lesions, where *NLRP3*-driven inflammation may dominate [43]. Studies in *ApoE*^-⁣/-^ mice suggest *CARD8* upregulation in advanced plaques reduces IL-1β, but its function in early endothelial dysfunction remains underexplored [135]. Second, sex-specific differences in *CARD8* regulation, such as the rs2043211 polymorphism’s impact on reducing inflammatory markers (e.g., Chemokine (C-C motif) ligand 20 (CCL20), IL-6) in males but not females, potentially due to estrogen-mediated effects, warrant further investigation to tailor therapies for diverse populations [135]. Third, evaluating combination therapies integrating *CARD8* modulation with statins could enhance efficacy; preclinical data on *NLRP3* inhibitors like MCC950 show synergistic reductions in plaque size when combined with atorvastatin, suggesting *CARD8* agonists could similarly augment lipid-lowering and anti-inflammatory effects [150]. These priorities—stage-specific functions, sex differences, and combination strategies—require targeted studies, including longitudinal human cohorts and genetic models, to validate *CARD8*’s clinical applicability and optimize personalized treatment approaches [46].

## Abbreviations

CAD, coronary artery disease; LDL, low-density lipoprotein; *CARD8*, Caspase Recruitment Domain Family Member 8; CARD, caspase recruitment domain; ox-LDL, oxidized LDL; Tregs, regulatory T cells; PAMPs, pathogen-associated molecular patterns; DAMPs, damage-associated molecular patterns; MCP-1, monocyte chemoattractant protein-1; DC, dendritic cell; IHC, immunohistochemical; BiKE, Biobank of Karolinska Endarterectomies; DAB, 3,3^′^-diaminobenzidine; MACE, major adverse cardiovascular events.
